# Fluid activity detection in geothermal areas using a single seismic station by monitoring horizontal-to-vertical spectral ratios

**DOI:** 10.1038/s41598-021-86775-1

**Published:** 2021-04-16

**Authors:** Kyosuke Okamoto, Hiroshi Asanuma, Hiro Nimiya

**Affiliations:** 1 Fukushima Renewable Energy Institute, National Institute of Advanced Industrial Science and Technology, 2-2-9, Machiikedai, Koriyama, Fukushima 963-0298 Japan; 2 Research Institute of Earthquake and Volcano Geology, National Institute of Advanced Industrial Science and Technology, 1-1-1, Higashi, Tsukuba, Ibaraki 305-8567 Japan

**Keywords:** Seismology, Geophysics, Natural hazards

## Abstract

Subsurface structure survey based on horizontal-to-vertical (H/V) spectral ratios is widely conducted. The major merit of this survey is its convenience to obtain a stable result using a single station. Spatial variations of H/V spectral ratios are well-known phenomena, and it has been used to estimate the spatial fluctuation in subsurface structures. It is reasonable to anticipate temporal variations in H/V spectral ratios, especially in areas like geothermal fields, carbon capture and storage fields, etc., where rich fluid flows are expected, although there are few reports about the temporal changes. In Okuaizu Geothermal Field (OGF), Japan, dense seismic monitoring was deployed in 2015, and continuous monitoring has been consistent. We observed the H/V spectral ratios in OGF and found their repeated temporary drops. These drops seemed to be derived from local fluid activities according to a numerical calculation. Based on this finding, we examined a coherency between the H/V spectral ratios and fluid activities in OGF and found a significance. In conclusion, monitoring H/V spectral ratios can enable us to grasp fluid activities that sometimes could lead to a relatively large seismic event.

## Introduction

Horizontal-to-vertical (H/V) spectral ratio of seismic ambient noise is known as an indicator of subsurface structures. Originally, the H/V spectral ratio was proposed as a seismic response approximate of subsurface S-wave structures known as the Nakamura method^1^. In this method, the H/V spectral ratio observed on the ground surface is considered as the resonance frequency and amplification of ground motions. In other words, the H/V spectral ratios are considered as the S-wave transfer function between the ground surface and bedrock. The fundamental idea of the Nakamura method was that P-wave (vertical component) motion was not largely affected by sedimentary layers while S-wave (vertical component) motion was amplified within the surface layers. H/V spectral ratios observed on soft grounds such as sedimentary layers show higher values whereas those on bedrocks are flat (equal to 1). There is another point of view to utilize H/V spectral ratios in which it is considered an aspect ratio of the elliptic orbit of Rayleigh waves. At this point, S-wave velocity structures can be estimated from H/V spectral ratios based on the Rayleigh wave theory^2^. Although there is a fundamental difference between these two approaches, H/V spectral ratios are a function of subsurface structures in either approach.

Spatial variation of H/V spectral ratios derived from various subsurface structures (e.g., irregular structures, slopes, anomalies) has been reported by many studies^3–6^. It is said that, in terms of the horizontal extent, H/V spectral ratios roughly affect the subsurface structure within one wavelength of the focused frequency band^7^. Therefore, spatially dense observations of H/V spectral ratios can reveal local variations in subsurface structures.

On the other hand, temporal changes of H/V spectral ratios have been hardly reported, although there are areas where temporal variations in subsurface structures are likely expected, that is, areas abundant in subsurface fluid flow. For example, temporal variations in elastic wave velocities in geothermal areas induced by natural earthquakes as well as operations of fluid extraction and injection have been reported^8,9^. Similar reports have been made on carbon dioxide capture and storage sites^10^. Fluid flows may play a key role in making the elastic wave velocity fluctuate. In this study, we considered that the existence of fluid activities tends to decrease the elastic wave velocity. However, the behavior of elastic wave velocities of porous media is complex because it is a function of effective stress (confining pressure minus pore pressure), porosity, saturation, pore fluid (e.g., water, vapor, gas, and air), temperature and other factors that change matrix parameters like chemical reactions.

**Figure 1:**
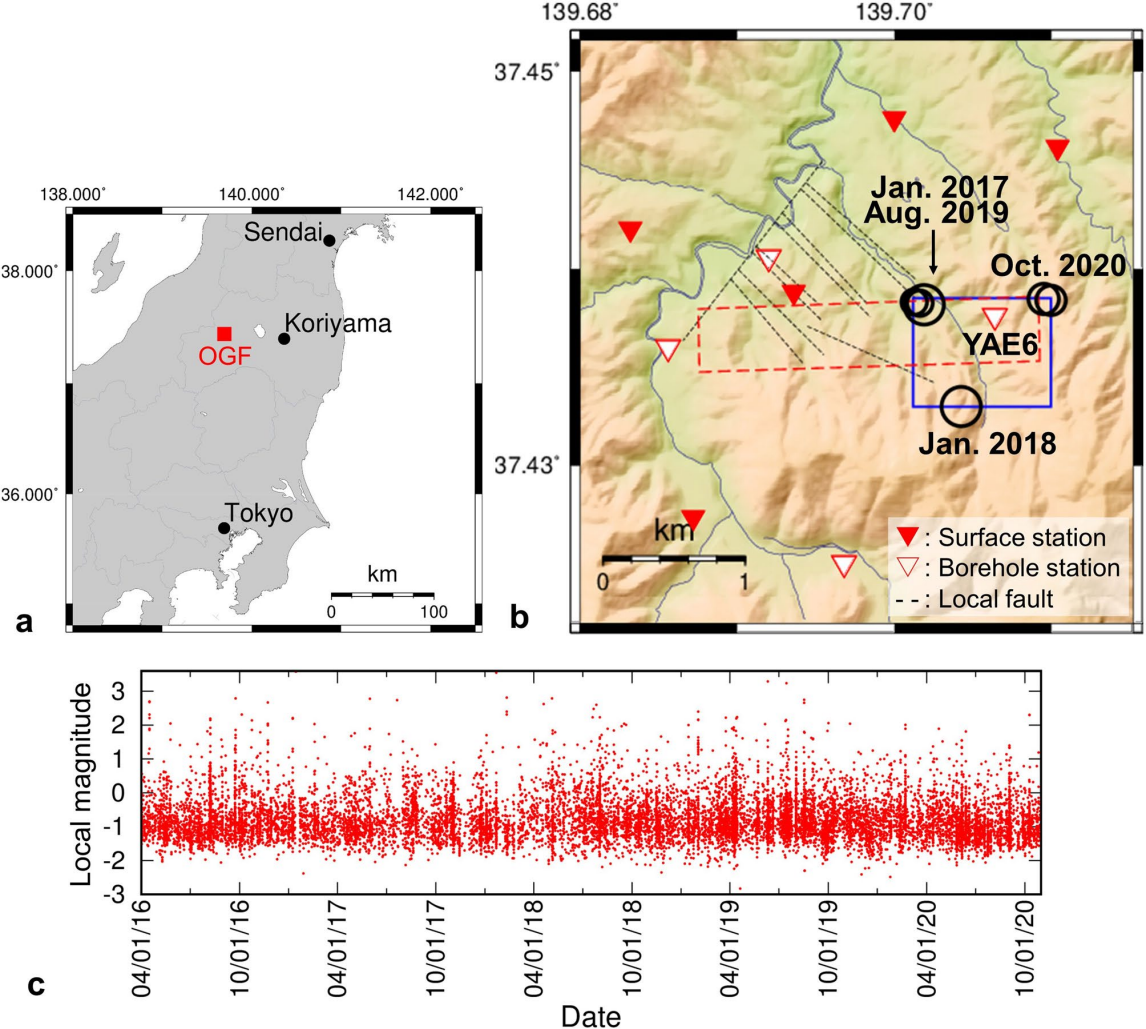
Overview of location, geometry, and seismicity of Okuaizu Geothermal Field (OGF). (**a**) Location of OGF on the main island of Japan. (**b**) Local geometry of OGF. The red dashed and blue solid rectangles show the areas for the numerical calculation of ambient noise propagation and the ETAS analysis, respectively. (**c**) Time history of seismic events was from April 1, 2016, to October 30, 2020.

In Okuaizu geothermal field (OGF), Japan, located in a volcanic front in the western part of Fukushima Prefecture (Fig. 1a), there is active microseismicity, which is considered to be related to fluid flows within and around the geothermal reservoir^9,11^. Since 2015, the Japan Oil, Gas and Metals National Corporation (JOGMEC) has been conducting gravity‐driven water‐injection tests into the reservoir to prevent the reduction of steam production^12^. A high-sensitivity seismograph network, composed of four and five sensors in the borehole and on the surface, respectively, has been deployed in OGF to monitor daily microseismic events (Fig. 1b). More than 14,000 seismic events were observed by the network from April 2016 to October 2020 (Fig. 1c). One of the borehole stations named YAE6 (the sensor is F41‐15.0, International Earth Sciences IESE Ltd.) has been installed at a 340-m depth apart from the production area of the geothermal reservoir and is located in a seismically active region around the Sarukurasawa Fault, where seismic events intensely occur regardless of the water injection. In this seismically active region, a seismic event with a magnitude of 4.9 occurred on October 12, 2009 (before the water injection test), which was concluded as a natural earthquake independent of the geothermal development^13^. There were four seismic events with local magnitude (M_L_) ≥ 2.7 in this region since 2016 (the circles in Fig. 1b): M_L_ 3.6 (January 13, 2017), M_L_ 3.6 (January 21, 2018), M_L_ 2.7 (August 17, 2019) and M_L_ 2.8 (August 17, 2019). In addition, two seismic events with M_L_ 2.3 occurred on October 9, 2020, within several seconds of each other at almost the same location. We considered these two seismic events as one seismic event with M_L_ 2.5. In total, there were five seismic events with M_L_ ≥ 2.5 since 2016, which seemed to occur regardless of the water injection operation.

**Figure 2:**
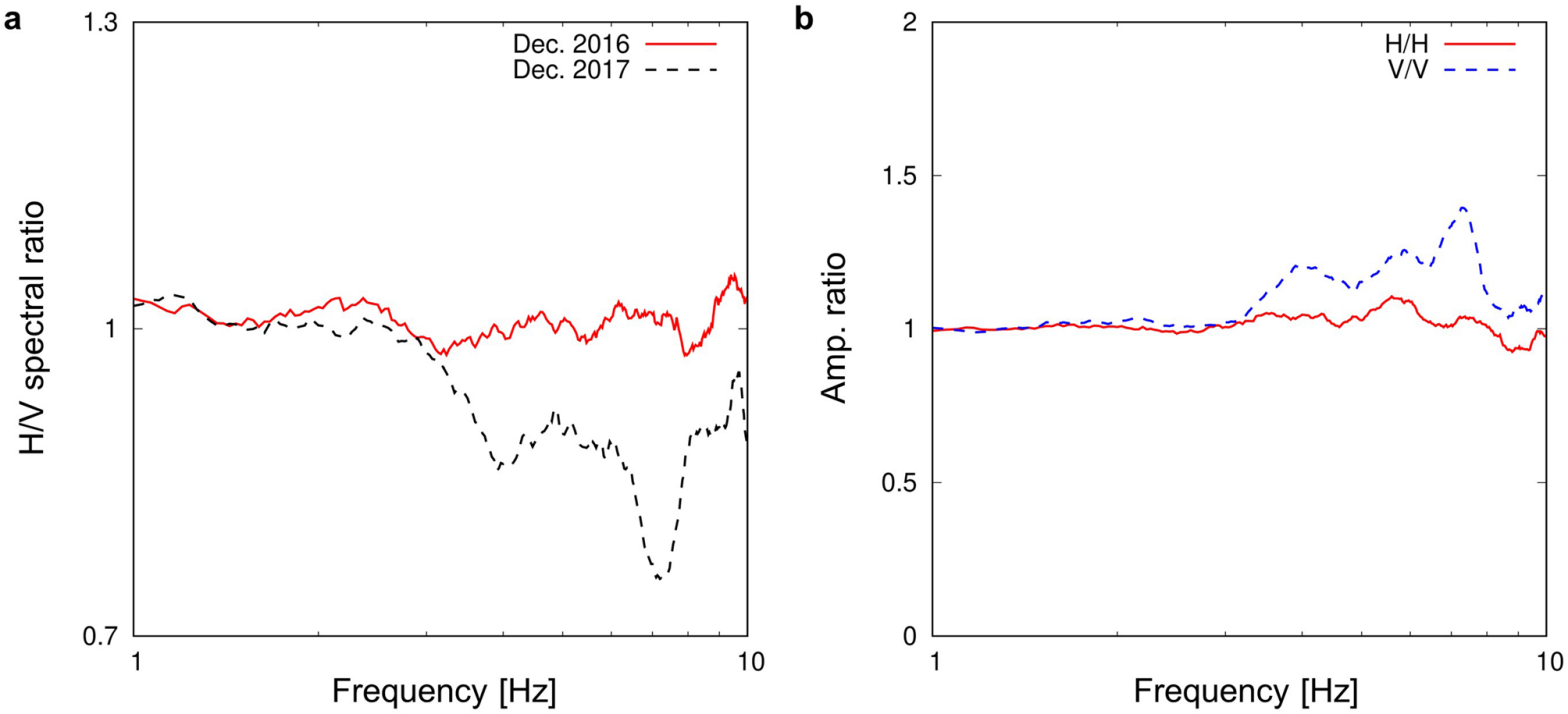
Temporary changes in the characteristics of seismic waves observed at YAE6. (**a**) The red solid and black dashed lines are the H/V spectral ratios of a day in December 2016 and a day in December 2017, respectively. (**b**) Amplitude ratios of seismic waves between the days in (**a**) (December 2017 over December 2016) are shown. The red solid and blue dashed lines are the results for the horizontal and vertical components, respectively.

Normally, H/V spectral ratios observed at YAE6 are almost flat over a 1–10 Hz band as shown in the red solid line in Fig. 2a, suggesting that YAE6 is located in hard rock according to the Nakamura method. However, we found that the H/V spectral ratios sometimes drop (the black dashed line in Fig. 2a) and recover after a while. One series of the drop and recovery took several days to months. The cause of the drop is temporary higher amplification in the vertical component of seismic waves compared to that in the horizontal component (Fig. 2b). We considered that this temporary amplification of seismic waves was derived from a decrease in elastic wave velocities, which is related to local fluid activities beneath YAE6.

In this study, firstly, we numerically examined the relationship between the H/V spectral ratio drop and the decrease in elastic wave velocities based on a finite difference method. We employed a 2-D elastic wave velocity structure^11^ within the red dashed line in Fig. 1b that was estimated by the double-difference seismic tomography method^14^. Secondly, we monitored the occurrence of a temporary drop in H/V spectral ratios at YAE6 from 2016 to 2020. Based on the result, we discussed the relationship with fluid activities beneath YAE6. If the seismic events along the Sarukurasawa Fault are directly or indirectly induced by fluid activities, monitoring the temporary drop in H/V spectral ratios observed by YAE6 can enable us to grasp or perhaps anticipate seismic activities. It could lead to evaluating immediate seismic risk.

## Results

### Numerical examination for drop of H/V spectral ratios

**Figure 3:**
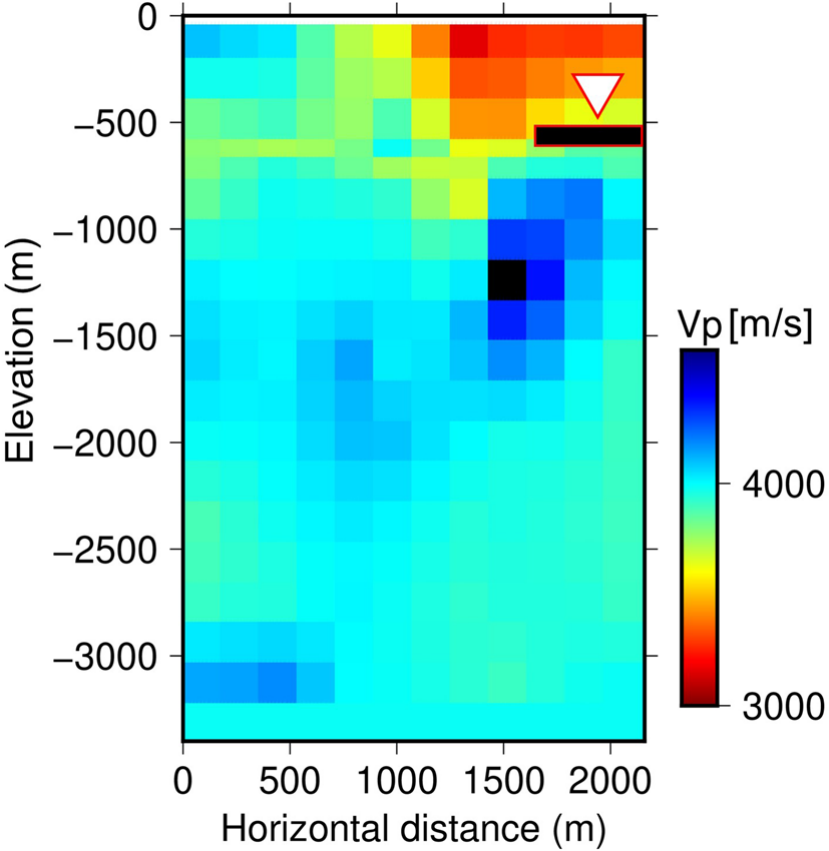
Elastic wave velocity structure estimated by the double-difference tomography method. The white triangle is YAE6. The red rectangle filled with black is the location of local fluid flows.

**Figure 4:**
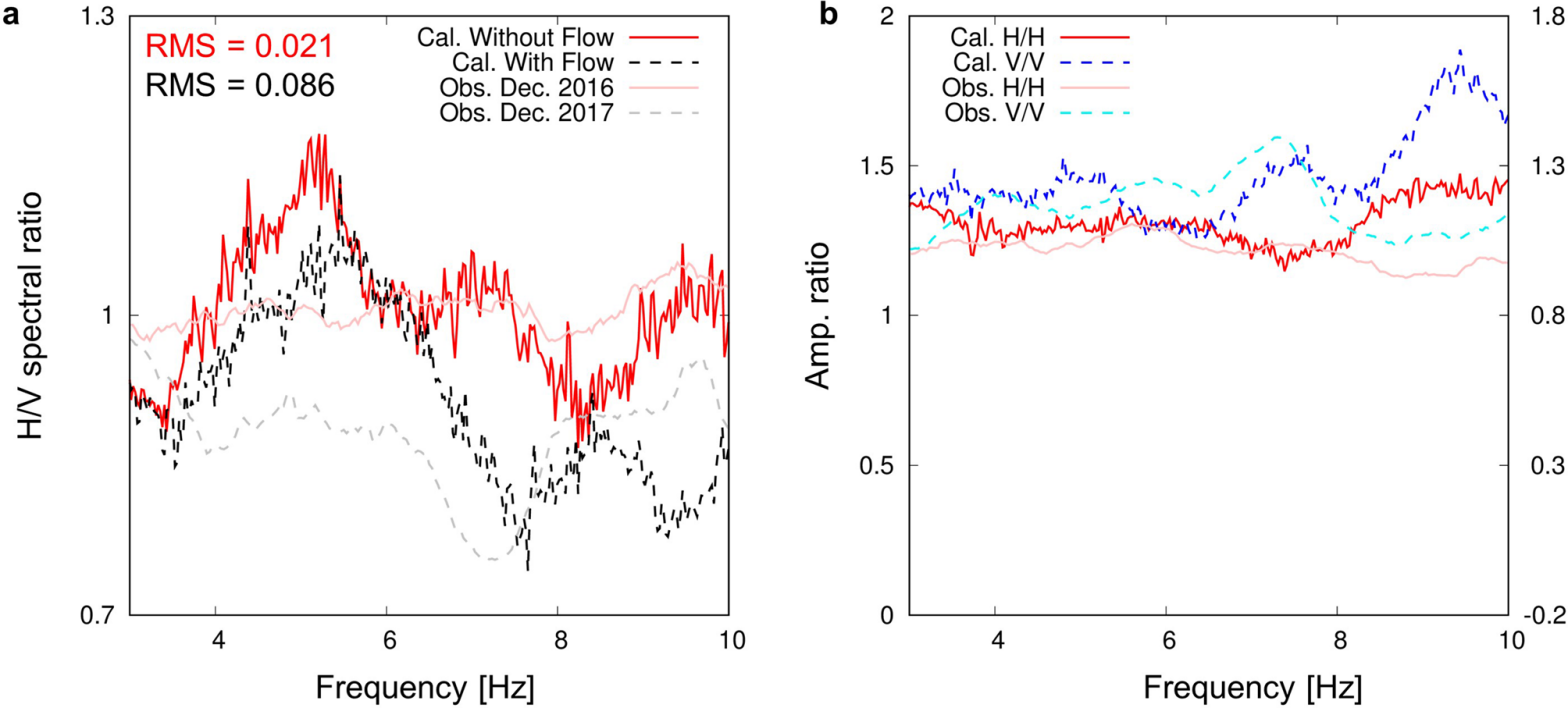
Numerical results seismic wave characteristics observed at YAE6. (**a**) The red solid and black dashed lines are the H/V spectral ratios for the cases without and with the low-velocity zone, respectively. The thin pink and gray lines are the observed H/V spectral ratios shown in Fig. 2a. (**b**) The amplitude ratios of seismic waves between the cases with and without the low-velocity zone (“with low-velocity zone” over “without velocity zone”) are shown. The red solid and blue dashed lines are the results for the horizontal and vertical components, respectively (the left vertical axis). The thin pink and blue lines are the observed ratios shown in Fig. 2b (the right vertical axis).

Based on the numerical calculation of ambient noise propagation in the model shown in Fig. 3, H/V spectral ratios were observed at the receiver in a borehole that simulated YAE6. The employed velocity structure was based on the double-difference seismic wave tomography conducted in the preceding study^11^. An initial geological survey at the early stage of developing OGF in 1970 to the 1980s estimated that an andesite rock reservoir was expanded below 1200-m deep (approximately 1600 m below sea level) and a shallow mudstone layer approximately 500-m deep (approximately 900 m below sea level) acted as cap zone^15^. The reservoir was strongly controlled by vertical fault zones, and hot water convection was estimated within these zones^15^. In our numerical model (Fig. 3), the reservoir area corresponds to the western area (approximately 0–1300 m in the horizontal distance). A 100-m thick low-velocity zone shown by the red rectangle filled with black in Fig. 3 (P-wave velocity (*V*_*P*_) = 2090 m/s, S-wave velocity (*V*_*S*_) = 700 m/s, density (*ρ*) = 1960 kg/m^3^: the relationships between those parameters were based on the Nafe–Drake empirical relation)^16^, which simulated a local fluid flow area in fractures, was located 160 m beneath the receiver. Figure 4a shows the H/V spectral ratios calculated for the cases with and without the low-velocity zone. The H/V spectral ratio for the case with the low-velocity zone (the black dashed line in Fig. 4a) was smaller than that for the case without the low-velocity zone (the red line in Fig. 4a) around a 4–10 Hz band. Moreover, a deep trough around a 6–8 Hz band appeared for the case with the low-velocity zone (the black dashed line in Fig. 4a), which was not seen for the case without the low-velocity zone (the red line in Fig. 4a). Root mean square (RMS) values for the fitting between observed and calculated curves (6–8 Hz band) are 0.021 and 0.086 for the cases without and with the low-velocity zone, respectively. Figure 4b shows amplitude ratios of seismic waves for the case with the low-velocity zone to those for the case without the low-velocity zone. In the former, both the horizontal and vertical components of seismic waves were amplified compared to the latter. Especially, the vertical component showed relatively larger amplification than the horizontal component and peaked around the 6–8 Hz band, unlike with the horizontal component. These characteristics decreased and troughed in the H/V spectral ratios for the case with the low-velocity zone (the black dashed line in Fig. 4a). Our calculation results can qualitatively explain the observed temporary drop in H/V spectral ratios (Fig. 2a or shown by the thin lines in Fig. 4a) as well as the amplification of seismic waves (Fig. 2b or shown by the thin lines in Fig. 4b), suggesting that temporary local fluid flows beneath YAE6 might occur repeatedly making the H/V spectral ratios drop.

We conducted numerical simulations by changing settings (elastic wave velocity and depth, see Supplementary Table S1) for the low-velocity zone. According to the results, low-velocity zone with lower (*V*_*P*_ 1510 m/s) or higher (*V*_*P*_ 2570 m/s) velocities than the original setting could still account for the observed temporary drop in H/V spectral ratios (RMS < 0.1), although deeper low-velocity zone did not make H/V spectral ratios drop (RMS > 0.1) (Supplementary Fig. S1). It seems that the depth of the low-velocity zone is an important factor determining whether the drop in H/V spectral ratios appears or not.

### Temporal changes of H/V spectral ratios at YAE6

**Figure 5:**
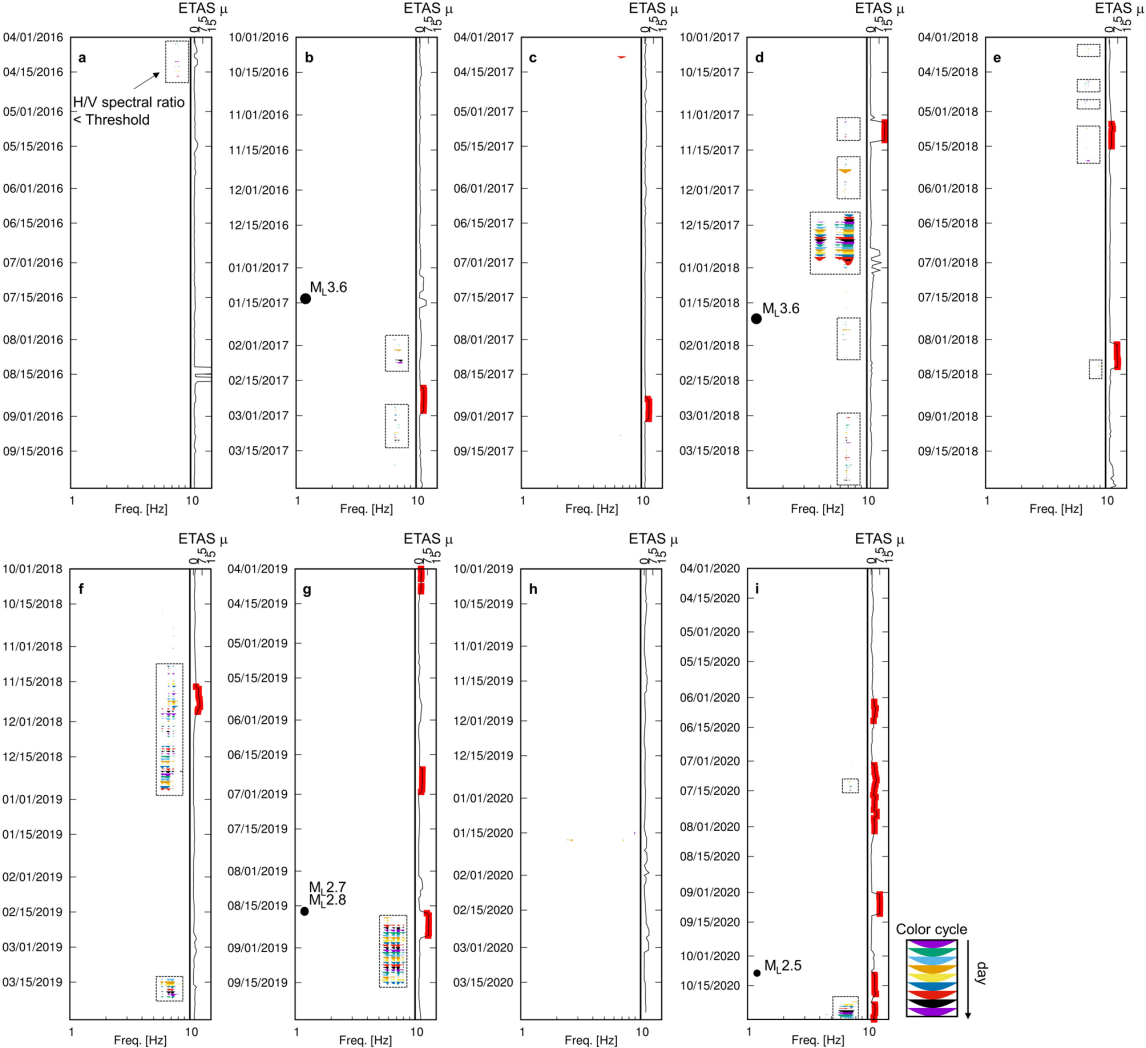
Monitoring results of low H/V anomalies and the μ value in the ETAS analysis. The low H/V anomalies are shown in the left column by the different colors, day by day. Swarms of the low H/V anomalies are indicated by dashed rectangles. The major seismic events shown in Fig. 1b are the black circle also in the left column. The curve of the μ values in the right column is remarked red if the condition μ  ≥ 2 is satisfied for a week or more.

We observed the temporal variation in H/V spectral ratios from April 2016 to October 2020. The parts of the H/V spectral ratios less than the threshold (0.87) were extracted, and the relation to subsurface fluid flows was examined (Fig. 5). The threshold of 0.87 was chosen not to overlook the temporal decrease in H/V spectral ratios shown in Fig. 4a. We employed the μ value in the epidemic type aftershock sequences (ETAS) analysis ^17^ as a proxy of subsurface fluid flows. The ETAS analysis is an appropriate tool for extracting the primary fluid signal from background natural seismicity ^18–21^. The μ value describes the number of seismic events for a day, which does not follow Omori’s law (thus, the number of seismic events was likely triggered by fluid flows). The curves of the μ value where remarked red in Figs. 5b–g, and Fig. 5i indicate the active fluid flow terms that we defined by the conditions below; the condition “μ  ≥ 2” continues longer than a week. Major seismic events (M_L_ ≥ 2.5) near YAE6 are indicated by the black circles in Fig. 5.

The H/V spectral ratios less than the threshold (hereafter, denoted as low H/V anomaly) occurred intermittently. Each low H/V anomaly continued for several days to several months. The first low H/V anomaly in the analysis term occurred around April 2016 (Fig. 5a). After that, low H/V anomalies did not appear for the following nine months. The second low H/V anomaly occurred around February 2017 with the active fluid flow term (shown by the red curve in Fig. 5b) after a seismic event with M_L_ 3.6 in January. From April to September 2017, there were no intense swarms of low H/V anomalies but a few short-term low H/V anomalies (Fig. 5c). In contrast, several swarms of low H/V anomalies were found over the following six months (October 2017–March 2018, Fig. 5d). Low H/V anomalies started together with the active fluid flow terms around the beginning of November 2017. The amplitude of low H/V anomalies increased in intensity from the middle of December 2017, then, the activity of low H/V anomalies suddenly diminished at the end of December. After this diminishment, a seismic event with M_L_ 3.6 occurred. Another swarm of low H/V anomalies was created from March 2018, which ended with the appearance of active fluid flow terms in May 2018 (Fig. 5e). In the middle of August 2018, a minor swarm of low H/V anomalies accompanied with the active fluid flow terms was found (Fig. 5e). From October 2018 to September 2019 (Fig. 5f,g), there were three major swarms of low H/V anomalies (November–December 2018, March 2018, and August–September 2019). Especially, the first and last swarms appeared with the active fluid flow terms. Seismic events with M_L_ 2.7 and 2.8 occurred right before the beginning of the last swarm. In the following six months (from October 2019 to March 2020), only a few low H/V anomalies appeared, and there were no active fluid flow terms (Fig. 5h). In the last eight months of the observation term (from April to October 2020, Fig. 5i), minor and major swarms of low H/V anomalies were found in July and October, respectively. Both were accompanied by the active fluid flow terms. A seismic event with M_L_ 2.5 occurred before the beginning of the latter swarm.

## Discussion

We pointed out the relationship between the low H/V anomalies and the active fluid flow terms defined by the μ parameter in the ETAS analysis. The swarms of low H/V anomalies tended to occur together with the active fluid flow terms. To discuss the significance of this relationship, we applied Fisher’s exact test for the cross-tabulation table of the numbers of the low H/V anomalies and active fluid flow terms. To count these numbers, we employed a 2-day long window with moving intervals of 2 days over the whole analysis term (April 2016–October 2020) (Table 1). The P-value of the Fisher’s exact test (two-sided test) was 1.4 × 10^–5^ (< 0.05); that is, the probability of occurrence of low H/V anomalies significantly increased when active fluid flows occurred (i.e., when the condition μ ≥ 2 was continued). Therefore, the monitoring of low H/V anomalies could indicate that the possibility of the existence of fluid activities near YAE6 is temporary increasing.

**Table 1:** Cross-tabulation regarding the active fluid flow term and low H/V anomaly.

	Low H/V anomaly appeared	Low H/V anomaly not appeared	All-time windows
Active fluid flow terms (μ ≥ 2 continued)	36	56	92
Inactive fluid flow terms (μ < 2 included)	136	609	745
All-time windows	172	665	837

**Figure 6:**
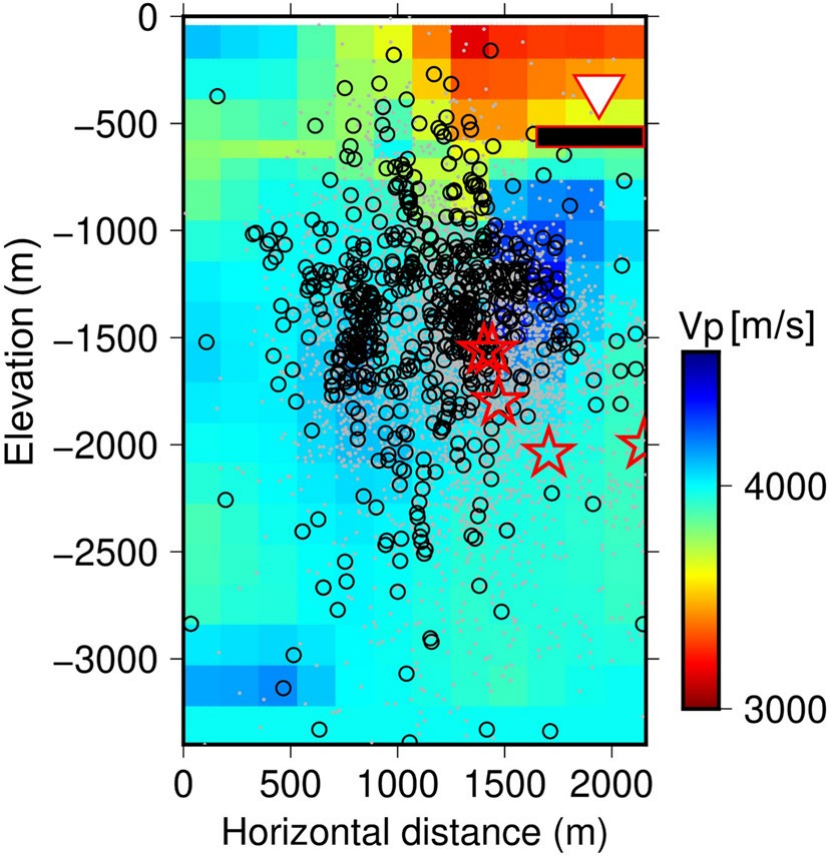
Seismic events from May 1, 2015, to October 2020 (gray dot). Seismic events from May 1, 2015 to May 30, 2016, which are precisely relocated by the double-difference tomography method^11^ are shown in black circles. The red stars indicate the major seismic events shown in Fig. 1b. Those seismic events are overlaid in Fig. 3.

On the other hand, we should note that the low H/V anomalies did not always occur with the active fluid flow terms, and the active fluid flow terms appeared sometimes without the low H/V anomalies. An inconsistency factor is that our target frequency (approximately 4–10 Hz, of which we expected low H/V anomalies) is sensitive to the velocity changes within several hundred meters beneath YAE6 (indicated by the red rectangle filled with black in Fig. 3) according to the numerical calculation. Therefore, the low H/V anomalies may not react to fluid flows deeper than those depths. On the other hand, most seismic events (which were determined by the double-difference seismic tomography method^14^ around YAE6) occurred at deeper depths than the sensitive depths of low H/V anomalies (Fig. 6). Consequently, the μ value in the ETAS analysis, which we adopted as a proxy of fluid flows, was mainly sensitive to the deeper depths. The difference in sensitive depths between the low H/V anomalies and μ value might lead to the inconsistency of their occurrence timing. However, it is noteworthy that the statistical test supported their mutual relation. We considered that fluid flows between their sensitive depths were invisible by seismic events and tied the low H/V anomalies and μ value activities. In OGF, it was reported that a seismic gap corresponded to the high peaceable path through which fluid passed without seismicity^11^. Another evidence shows that the low H/V anomalies are sensitive to the changes in condition around the receiver. Temporal increase in elastic wave velocity beneath YAE 6 estimated by single-station cross-correlation technique for passive seismic waves^22–24^ were accompanied by the swarm of the strong low H/V anomalies that appeared in the middle of December 2017 (Supplementary Fig. S2). More details on the method for estimating temporal elastic wave velocity changes are found in the supplementary material.

Monitoring the low H/V anomalies could have a substantial potential to estimate immediate seismic risk. In the analysis term, there were five seismic events with M_L_ ≥ 2.5, and all were preceded or followed by the low H/V anomalies (Fig. 5). The time gaps between those seismic events and the low H/V anomalies were approximately 10 days at most. The seismic events with M_L_ 3.6 on January 21, 2018, were within the swarm of low H/V anomalies. In the case of the seismic events with M_L_ 2.7 and M_L_ 2.8 on August 17, 2019, the following low H/V anomalies appeared a day later. Perhaps, monitoring precursory fluid and post-seismic fluid activities related to relatively large-sized seismic events that could allow earthquakes be felt can be achieved. A major merit of this monitoring method is the convenience and low-cost of deploying and maintaining the monitoring network (at least one borehole sensor near the focused area). A limitation of this study was the lack of an adequate number of the relatively large-sized seismic events in OGF to examine the availability of the monitoring method. A much longer observation period in OGF and/or further case studies in other fields are needed to verify the availability and develop the monitoring procedure.

## Conclusions

In this study, we found a temporary drop in H/V spectral ratios in a geothermal area in Japan. We revealed that the drop in H/V spectral ratios could be accounted for by the decrease in elastic wave velocities derived from fluid activities beneath the receiver based on a numerical calculation. Therefore, we considered that monitoring the temporary drop in H/V spectral ratios could illuminate subsurface fluid activities, and we could anticipate a relatively higher seismic event if it is a product of the fluid activities. Further studies in other fields as well as in OGF are needed to reveal much detailed investigation of the low H/V anomaly (e.g., applicability to other fields, correlation with the evidence of fluid flows other than the μ value, and examination of the possibility to use other criteria obtained from seismic data).

## Methods

### Numerical calculation of ambient noise field

We employed a 2-D finite difference method in the time domain with a rotated staggered grid^23^, which can accurately calculate seismic wave propagation in media with high-impedance contrast, such as the interfaces between air, fluid, and rock. An absorbing boundary condition^25^ was introduced to the lateral and bottom boundaries. White noise was inputted to normal stress components of 96 points, which were randomly selected from the space between the surface and a depth of 60 m. The white noise was synthesized from the frequency spectra, which have the same amplitude and random phases against the frequency using a fast Fourier transform. A 46-s long record was synthesized. Detail of the calculation is described in the preceding studies^7,26^.

### Calculation of H/V spectral ratios from continuous observed data

We employed a 30-s long time window to calculate H/V spectral ratios. This time window was applied to every half hour daily. Therefore, 48 H/V spectral ratios were estimated a day and were averaged to obtain the value of each day. The sampling rate of the continuous seismic waves is 1000 Hz.

### ETAS analysis using moving time window

In the ETAS model, each seismic event was assumed to produce aftershocks that follow Omori’s law. The entire seismicity is described by the superposition of the aftershocks, as well as seismic events that do not follow Omori’s law. Especially in geothermal areas where rich fluid flows exist, the latter seismic events seem to be derived from fluid activities^27^. The number of those seismic events a day was described by the μ value in the ETAS analysis. We estimated the μ value using a 10‐day time window that moves at intervals of one day. The estimated μ value was adopted as the value for the last day of the time window coverage. We only used the seismic events that occurred within the blue rectangle shown in Fig. 1b to focus on fluid activities near YAE6. In the ETAS analysis, it is needed to define the start of the precursory period of seismic activity that precedes the time window. May 1, 2015 was set as the start of the precursory period when the routine hypocenter determination begun in OGF. Notably, the sensor of YAE6 that had been installed till January 2016 lacked heat tolerance and was not qualified to conduct the H/V spectral ratio analysis. In this study, we used seismic wave data of YAE6 only after replacing the current sensor with enough heat tolerance.

## Supplementary Information

Supplementary Information.

## Data Availability

JOGMEC has proprietary use of the microseismic data. Details of the data (continuous row data, earthquake catalog) are available from the corresponding author upon request with the approval of JOGMEC.
