# Supplementary material for: Fluid activity detection in geothermal areas using a single seismic station by monitoring horizontal-to-vertical spectral ratios
Source: Sci Rep. 2021 Apr 16;11:8372. doi: 10.1038/s41598-021-86775-1 (PMC8052333; doi:10.1038/s41598-021-86775-1)
Supplement: Supplementary file 1 — Supplementary Information. [file 41598_2021_86775_MOESM1_ESM.pdf]

Supplementary information

Fluid activity detection in geothermal areas using a single seismic station by monitoring horizontal-to-vertical spectral ratios

Kyosuke Okamoto[1], Hiroshi Asanuma[1] and Hiro Nimiya[2]

1) Fukushima Renewable Energy Institute, National Institute of Advanced Industrial Science and Technology, 2-2-9, Machiikedai. Koriyama, Fukushima 963-0298, Japan.
2) Research Institute of Earthquake and Volcano Geology, National Institute of Advanced Industrial Science and Technology, 1-1-1, Higashi, Tsukuba, Ibaraki 305-8567 Japan.

Corresponding author: Kyosuke Okamoto (okamoto.kyosuke@aist.go.jp)

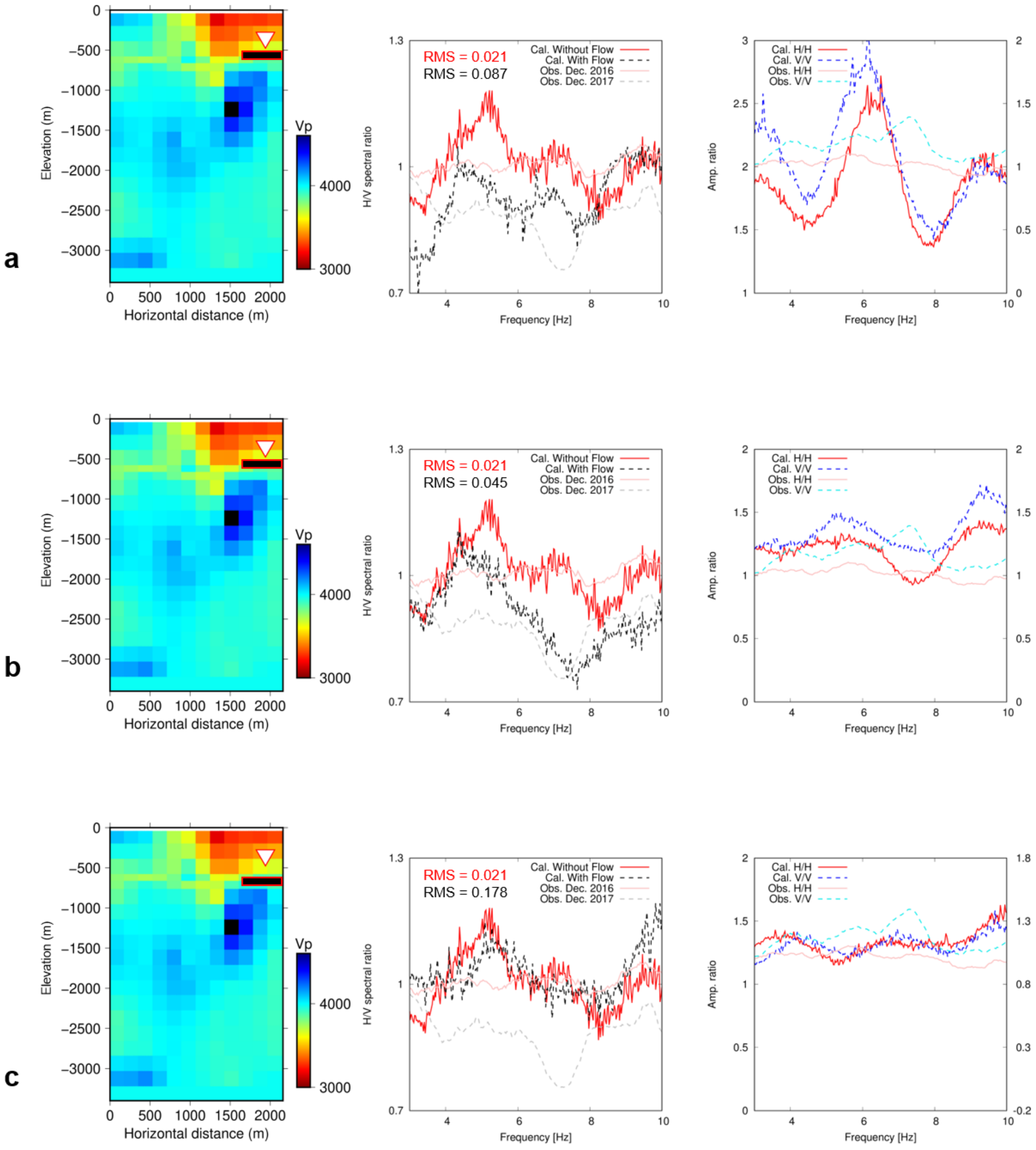
a
b
c
Elevation (m)
Horizontal distance (m)
Vp
4000
3000
RMS = 0.021
RMS = 0.087
RMS = 0.045
RMS = 0.178
Cal. Without Flow
Cal. With Flow
Obs. Dec. 2016
Obs. Dec. 2017
H/V spectral ratio
Frequency [Hz]
Cal. H/H
Cal. V/V
Obs. H/H
Obs. V/V
Amp. ratio

d

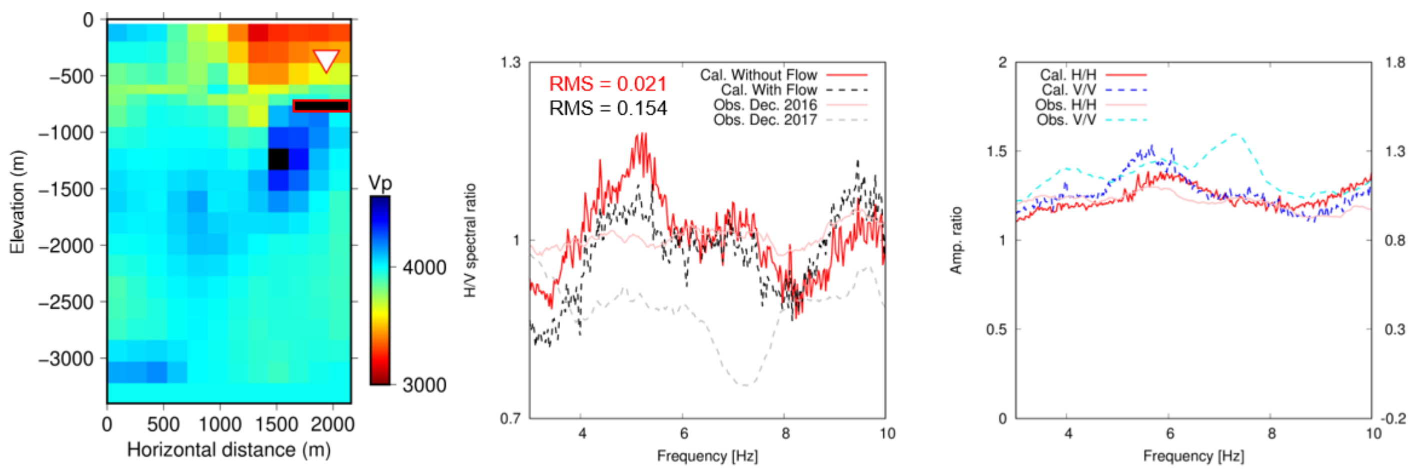

Figure S1. Numeral results for cases for various settings of velocity anomaly. (Left) Numerical models: (Center) Numerical results of H/V spectral ratios (the bold lines) with the real observed H/V spectral ratios in OGF (the thin lines): (Right) amplitude of seismic waves between the cases with and without the low-velocity zone. The detail of the figure legends corresponds to Figs 3 and 4 in the main content. Setting for the anomaly in the (a)–(d) cases is listed in Table S1.

Table S1. Settings of anomaly for numerical calculations

	P-wave (m/s)	S-wave (m/s)	Density (kg/m^3)	Depth beneath the receiver (m)
Original setting in the main content	2,090	700	1,960	160
Fig. S1a	1,510	50	1,570	160
Fig. S1b	2,570	1,200	2,130	160
Fig. S1c	2,090	700	1,960	260
Fig. S1d	2,090	700	1,960	360

We applied the single-station cross-correlation (SC) technique to monitor a seismic velocity change that reflects subsurface variations around the station. SC is the correlation of two different components of a single three-component seismic records[1–3]. We used three-component continuous records observed at station YAE 6 from April 1, 2016, to October 31, 2020. First, we carried out down-sampling from 1,000 to 50 Hz and divided 1-day data into 20-min segments with 50% overlap. Each segment was low-pass-filtered at 8 Hz. To remove the noisy data, we discarded the segments whose root-mean-square amplitude (RMS) exceeded a threshold. We defined the threshold as 1.2 times the mean value of RMSs for all the segments in 1 day. The SCs were computed in the frequency domain using the following equation

$$SC_{k1k2}(f) = \frac{u_{k1}(f)u_{k2}^{*}(f)}{|u_{k1}(f)||u_{k2}(f)|}, \quad \text{(S1)}$$

where SC is the SC function, f is frequency, u is the seismic records in the frequency domain, and k_1 and k_2 stand for the components Z (vertical), E (east-west) and N (north-south), but $k_1 \neq k_2$. We then computed daily SCs by stacking each segment in 1 day. Finally, daily SCs were transformed into time-domain after applying a bandpass filter between 4–8 Hz.

We applied the stretching interpolation technique to estimate velocity variation $\Delta v/v$ [4]. Assuming a spatially homogeneous velocity change, the arrival time change can be indicated by the following equation

$$\frac{\Delta t}{t} = -\frac{\Delta v}{v}, \quad \text{(S2)}$$

where t is the lag time of the SC and Δt is the time shift in the SC at t, and $\Delta v/v$ is a velocity change ratio. We stretch and compress a current waveform using predicted time delay and cross-correlate with a reference waveform. We can obtain the optimum value of $\Delta v/v$ by maximizing the cross-correlation coefficient between the current and reference waveforms. In this study, we used all-stacked and 15-day-stacked waveforms as the reference and current waveforms, respectively. The time window of 3–10 s was examined. The optimum value of $\Delta v/v$ was estimated by grid searches within the range from -1 to 1%, with steps of 0.001%. To stabilize $\Delta v/v$, we averaged the velocity changes of the different components. The estimated velocity change is shown in Fig. S2.

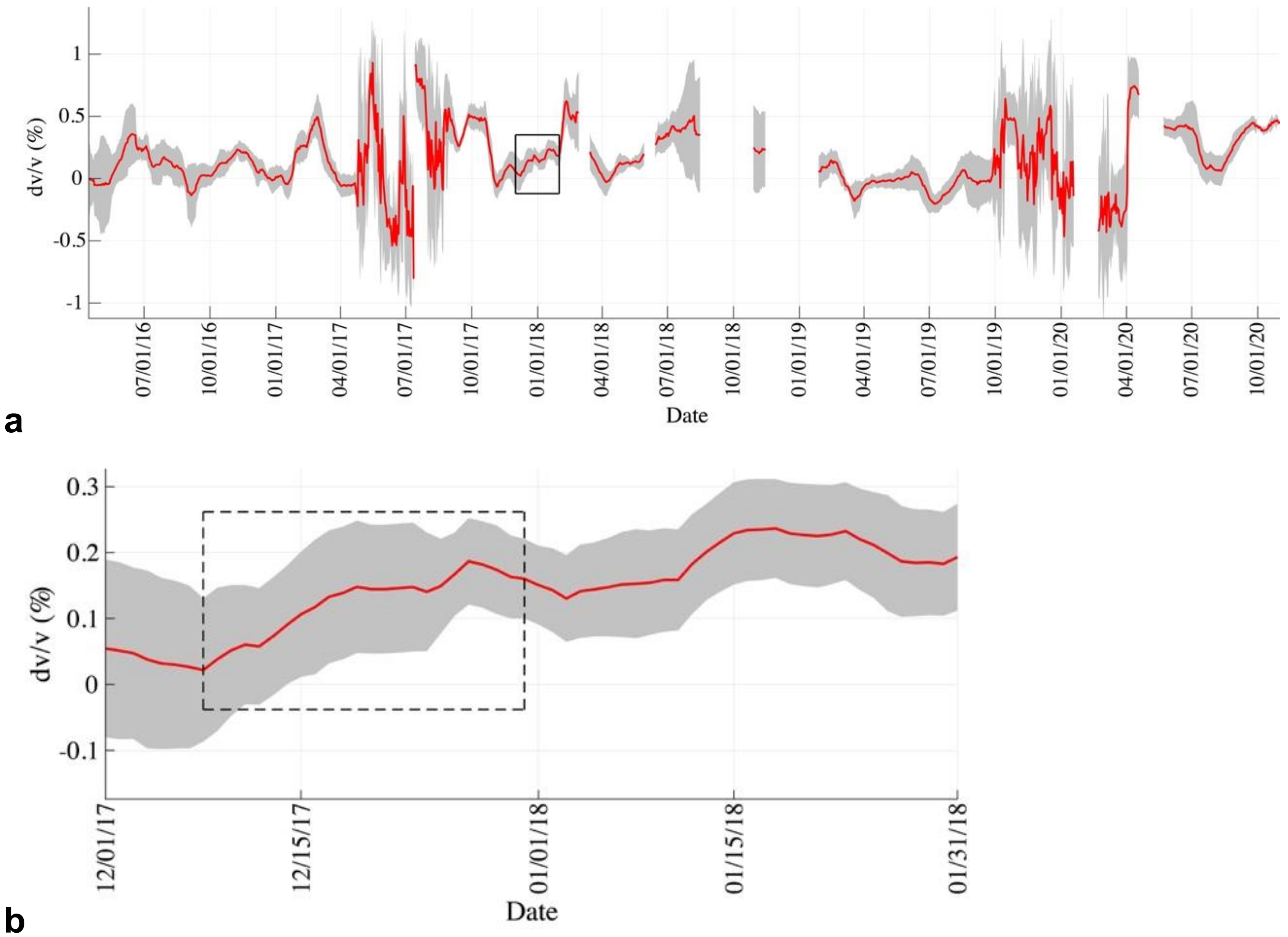

Figure S2. Estimated velocity change at the station YAE 6. The averaged velocity change is indicated by red lines. The gray shading indicates the standard deviation of the velocity change of 6 different components. (a) Result for the whole analysis term. (b) Enlarged view in the area indicated by the black rectangle in (a). The black dashed rectangle indicates the term with the swarm of the strong low H/V anomalies.

1. Hobiger, M., Wegler, U., Shiomi, K. & Nakahara, H. Single-station cross-correlation analysis of ambient seismic noise: Application to stations in the surroundings of the 2008 Iwate-Miyagi Nairiku earthquake. *Geophys. J. Int.* (2014). doi:10.1093/gji/ggu115
2. Viens, L., Denolle, M. A., Hirata, N. & Nakagawa, S. Complex near-surface rheology inferred from the response of Greater Tokyo to strong ground motions. *J. Geophys. Res. Solid Earth* (2018). doi:10.1029/2018JB015697
3. Sens-Schönfelder, C. & Wegler, U. Passive image interferometry and seasonal variations of seismic velocities at Merapi Volcano, Indonesia. *Geophys. Res. Lett.* (2006). doi:10.1029/2006GL027797
4. Hadziioannou, C., Larose, E., Coutant, O., Roux, P. & Campillo, M. Stability of monitoring weak

changes in multiply scattering media with ambient noise correlation: Laboratory experiments. *J. Acoust. Soc. Am.* (2009). doi:10.1121/1.3125345
